# Mid-term clinical results of primary total knee arthroplasty using metal block augmentation and stem extension in patients with rheumatoid arthritis

**DOI:** 10.1186/s12891-015-0689-9

**Published:** 2015-08-27

**Authors:** Satoshi Hamai, Hisaaki Miyahara, Yukio Esaki, Goh Hirata, Kazumasa Terada, Nobuo Kobara, Kiyoshi Miyazaki, Takahiro Senju, Yukihide Iwamoto

**Affiliations:** Department of Orthopaedic Surgery and Rheumatology, Clinical Research Institute, National Hospital Organization Kyushu Medical Center, 1-8-1 Jigyohama, Chuo-ku, Fukuoka 810-8563 Japan; Department of Orthopaedic Surgery, Faculty of Medical Sciences, Kyushu University, 3-1-1 Maidashi, Higashi-ku, Fukuoka 812-8582 Japan

## Abstract

**Background:**

Despite recent advancements in rheumatoid arthritis (RA) pharmacotherapy, surgeons still encounter severely damaged knees. The purpose of the present study was to analyze the mid-term clinical results of total knee arthroplasty (TKA) with metal block augmentation and stem extension.

**Methods:**

A total of 26 knees in 21 patients who underwent primary TKA with metal block augmentation and stem extension were retrospectively reviewed. All patients with a mean age of 63 years had RA for a mean duration of 15 years. Functional and radiographic results as well as complications were evaluated at the mean follow-up period of 6 years after TKA. Eight knees were lost follow-up after the two-year evaluation.

**Results:**

Tibial bone defects with average depth of 19 mm were preoperatively recognized in all 26 knees. The postoperative joint line was reconstructed on average 11 mm above the fibular head using average thickness of 11 mm tibial inserts and 9 mm metal blocks with stem extension. Significant improvements (*p* < 0.05 for all comparisons) were observed postoperatively in maximum extension angle from −10° to −1°, range of motion from 101 ° to 115 °, and Knee Society Score (knee score/function score) from 35/18 to 90/64. Non-progressive radiolucent lines beneath the metal block and osteosclerotic changes around the medullary stem were found in 16 knees (62 %) and 14 knees (54 %), respectively. There was two failures (8 %): fragile supracondylar femur fractures and knee instability. No knees showed any radiographic implant loosening, dislocation, polyethylene insert breakage, peroneal palsy, or infection.

**Conclusions:**

Primary TKA with metal block augmentation and stem extension could effectively restore function in RA patients with advanced forms of knee joint destruction, and be reliable and durable for a mean postoperative period of 6 years. Further study is needed to determine the long-term results of TKA using metal block augmentation and stem extension.

## Background

Total knee arthroplasty (TKA) provides excellent long-term results for pain relief and functional restoration in patients with rheumatoid arthritis (RA), osteoarthritis (OA), and other degenerative conditions of the knee joint [1, 2]. Despite recent advancements in RA pharmacotherapy, surgeons performing TKA still encounter severe cases of RA involving large bone defects, flexion contracture, severe valgus deformity, and other advanced forms of knee joint destruction. These deformities represent a major challenge in reconstructing bone deficiencies and achieving correct soft-tissue balancing.

The combination of bone graft transplantation or metal augmentation and stem extension is indicated for noncontained bone deficits greater than 15-mm deep from intact tibial articular surface [3]. Bone grafting of osseous defects, which assists in the restoration of bone stock, is recommended in relatively young patients. However, these patients require close follow-up because they may experience insufficient graft incorporation, osteonecrosis, and bone collapse [4, 5]. Metal augmentation can achieve early fixation, allowing for quicker rehabilitation after the surgery. Consequently, this approach is suitable for patients with compromised bone strength and elderly patients who require abbreviated post-operative hospitalization periods because of comorbidities requiring careful medical attention [6, 7].

Post-TKA complications in severely damaged RA knees could include implant loosening [8], residual restrictions in range of motion (ROM) [9], instability [10, 11], dislocation [12], peroneal palsy [11], infections [8, 13], and periprosthetic fracture [10, 13]. However, few studies have reported outcomes of TKA in severely degenerated RA knees [14]. The purpose of this study was to retrospectively evaluate mid-term postoperative clinical results of RA knees that underwent primary TKA with metal block augmentation and stem extension.

## Methods

From January 1999 to December 2010, 675 RA knees underwent primary TKA at the National Kyushu Medical Center, Fukuoka, Japan. All TKA surgeries were performed using the NexGen LPS, LPS-Flex, or LCCK prostheses (Zimmer, Warsaw, Indiana). Combination of metal block augments and stem extensions were employed in 31 knees (5 %) compensate for the peripheral defects of the tibia and included in this study. LCCK were implanted in 12 knees (2 %) to control varus-valgus instability and excluded from this study. Twenty one patients (84 %; 26 knees) who were followed up for 2 or more years postoperatively signed informed consent forms to participate in this retrospective study. The study was carried out after obtaining approval from medical ethics committee of our institution (IRB #12-06/National Hospital Organization Kyushu Medical Center).

The study population consisted of 18 females (23 knees) and 3 males (3 knees) with a mean age of 63 years (range, 47 to 74) at the time of surgery. The mean follow-up period was 6 years (range, 2 to 11.2). Six females (8 knees) are lost to follow-up after the two-year evaluation. All 21 patients had RA, for a mean duration of 14.6 years (range, 2.4 to 39). According to the Steinbrocker functional classification [15], 1, 7, 11, and 2 patients were rated as Classes I, II, III, and IV, respectively. The disease-modifying antirheumatic drugs that the patients were taking were methotrexate, sulfasalazine, bucillamine, and biologic agents. Sixteen patients (21 knees) were prescribed oral glucocorticoid with a mean dose of 7.7 mg (range, 1 to 15).

The surgeon made a midline longitudinal skin incision and exposed the knee joint with a medial parapatellar capsular approach using a pneumatic tourniquet. Intramedullary guides were used to cut the distal femur in 6 degrees of valgus relative to the anatomical axis and to cut the proximal tibia perpendicular to the tibial mechanical axis. The femoral rotational alignment was determined using the measured resection technique with reference to the epicondylar axis, Whiteside’s line, posterior condylar axis, and other anatomical landmarks. The tibial rotational alignment was established with reference to the tibial anteroposterior axis that connected the medial one-third of the tibial tuberosity and the center of the tibial insertion of the posterior cruciate ligament. For tibial metal augmentation, rectangular flat blocks were used in combination with 100-mm-long stem extensions. The surgeon carefully achieved proper medial and lateral ligament balancing. In varus knees, ligaments were detached sequentially after removal of osteophytes, starting from the medial side of the proximal tibia. For valgus knees, the surgeon only minimally detached the medial collateral ligament, and released the lateral supporting structures such as the iliotibial band and lateral fibers of the posterior joint capsule. In flexion contracture knees, the oblique rectus snip technique was applied, whereby the rectus tendon was cut at a 45-degree angle in conjunction with the medial parapatellar incision [16]. The femoral and tibial components were fixed with cement (Simplex P Bone cement: Stryker, Mahwah, New Jersey) to the cut surface by finger-packing without vacuum mixing or pulsed lavage, while the stem extensions were press-fitted without cement [17, 18]. Axial compressive load was applied in knee extension with a trial insert until the cement cures. A passive range of knee exercises and full weight-bearing on a caster walker started one or two days after the operation according to the extent of pain and general conditions of patients.

This study examined the following parameters: preoperative and postoperative ROMs, clinical scores according to the Knee Society Clinical Rating System [19], femorotibial angles (FTAs), postoperative femoral component coronal (α) and sagittal (γ) angles and tibial component coronal (β) and sagittal (δ) angles [19], joint line positions [20], sizes of metal block augmentations, tibial polyethylene inserts, and tibial stem extensions, presence of radiolucent lines and their ratings [19], radiographic findings of intramedullary osteosclerosis and heterogeneous cortical proliferation [21], and postoperative complications. The Anderson Orthopaedic Research Institute (AORI) classification system was used to evaluate the severity of bone loss [22], and the depths of the bone defect from intact tibial articular surface were quantitatively measured [3].

Changes in ROM (extension/flexion), Knee Society Score (KSS; knee score/function score), and FTA while standing before and after TKA were evaluated using the paired *t*-test. The cumulative probability of the revision TKA was estimated using the Kaplan-Meier product-limited method with 95 % confidence intervals (CI). For all statistical analysis, a significant difference was defined as a *P*-value < 0.05.

## Results

According to the AORI, type 2A tibial bone defects were recognized in all 26 knees and the mean depth of these tibial defects were 19 mm (range, 12 to 30). The postoperative joint line was located proximal to the top of the fibular head by a mean of 11 mm (range, 5 to 24). Metal blocks were used to compensate for the peripheral defects of the tibia in all 26 knees, including 25 knees (96 %) with medial condyle defects and 1 knee (4 %) with lateral condyle defects. The mean thicknesses of the polyethylene inserts and metal blocks used in these knees were 11 mm (range, 9 to 17) and 9 mm (range, 5 to 15), respectively. Tibial stem extensions had a mean diameter of 12.3 mm (range, 10 to 14) without offset in 14 knees (54 %) or with offset in 12 knees (46 %).

The mean preoperative and postoperative ROMs, KSS, and FTAs were described in Table 1. Four knees had severe flexion contractures beyond 30 degrees. In terms of knee alignment, 24 knees (19 patients) were varus, and 2 knees (2 patients) were valgus. Nine knees had severe varus deformities (FTA > 190 degrees), while 1 had severe valgus deformities (FTA < 160 degrees). After TKA, fifteen knees (58 %) had a ROM above 120 degrees. Statistical analyses revealed no significant difference pre- and postoperatively in maximum flexion angle, but significant improvements were observed postoperatively in maximum extension angle (*p* = 0. 001), ROM (*p* = 0.04), knee score (*p* < 0.0001), function score (*p* < 0.0001), and FTA (*p* < 0.0001). Component angulations were also described in Table 1. These angles showed no significant changes over time, up to the final observations.

**Table 1:** Clinical and radiographic data before and after TKA with metal block augmentation and stem extension in RA

Parameters	Preoperative values	Postoperative values
Extension/flexion angle (°)	−10 (0–30)/111 (30–140)	−1 (−5–0)* /116 (90–135)
KSS (knee score/function score)	35 (0–70)/18 (0–70)	90 (76–100)*/64 (5–100)*
FTA while standing in varus/valgus knees (°)	191 (179–204)/159 (153–164)	176 (172–179)*/175 (174–175)
Femoral component coronal (α) and sagittal (γ) angles (°)	N/A	93 (89–99)/0 (−5–3)
Tibial component coronal (β) and sagittal (δ) angles (°)	N/A	90 (88–92)/85 (81–91)

*TKA* total knee arthroplasty, *RA* rheumatoid arthritis, *FTA* femorotibial angle, *KSS* Knee Society Score, *N/A* not available

Values are given as mean (range), *significantly different between patients before and after TKA (*p* < 0.05)

On the femoral side, six knees (23 %) showed 7 non-progressive radiolucent lines (<2 mm) with 2 detected in zone 1 (anterior segment) and 5 in zone 4 (posterior segment). On the tibial side, 16 knees (62 %) had non-progressive radiolucent lines. In particular, 15 knees (58 %) showed radiolucent lines beneath the metal block (zones 1–2 or 3–4). However, the mean sum of radiolucent lines beneath in all zones of the tibial side was 1 mm (range, 0 to 4), and no knees had 5 mm or above. No significant changes were detected over time. Radiographic assessment around the medullary stems of the tibia showed osteosclerotic changes in 14 knees (54 %). Eleven knees showed osteosclerotic changes that ran parallel to and away from the stem surface, 2 knees showed osteosclerotic changes in contact with the stem, and 1 knee showed osteosclerotic change divergent toward the distal tip of the stem extension. Nine knees (35 %) showed radiolucent lines beneath the tibial component and intramedullary osteosclerosis, 7 knees (27 %) showed radiolucent lines only, and 5 knees (19 %) had osteosclerotic changes only. One subject (4 %) who showed radiolucent lines in the bilateral condyles also showed osteosclerotic lesions spreading toward the distal tip of the stem. However, these changes did not increase or spread over time. Four knees (15 %) showed heterogeneous cortical proliferation at the distal end of the tibial stem extension, although the lesions were not associated with pain.

Postoperative complications that necessitated surgical corrections occurred in 3 knees, including two revision TKAs (8 %). Specifically, the details of the 3 knees were as follows: 1 knee developed a fragility fracture of the femoral condyle 1 month postoperatively and underwent replacement of the previous femoral implant with a stem extension; 1 knee showed varus-valgus instability 2.5 years postoperatively and underwent revision with a thicker insert; and 1 knee had a supracondylar femoral fracture because of a fall, and was treated with intramedullary nail fixation. No knees showed any radiographic implant loosening, dislocation, tibial polyethylene insert breakage, skin necrosis, peroneal nerve palsy, or infection up to the final observations. Finally 18 knees remained for survival analysis, and Kaplan-Meier analysis with revision TKA as the end point showed a six-year survival rate of 91.6 % (95 % confidence interval, 71.7 to 97.9 %).

## Discussion

We performed a retrospective study of primary TKA using metal block augmentation and stem extension in RA patients with large bone defects. Most of the knees required metal blocks to compensate for deficiencies in the medial peripheral portions of the tibia from varus deformities, but only one knee (4 %) required metal block in the lateral side. The postoperative joint line was located on average 11 mm above the fibular head with proper alignment of the femoral and tibial components. Peripheral tibial deficits with a mean depth of 19 mm were reconstructed by metal blocks and tibial inserts with mean thicknesses of 9 mm and 11 mm, respectively. The clinical outcome was generally good, and the complication rate with revision TKA as failure was 8 %. There were one early and one late failures: a fragile supracondylar femur fracture which was revised with femoral stem extension and a case of knee instability which required polyethylene insert revision. No knees showed radiographic implant loosening, dislocation, peroneal palsy, or infection up to the final observations.

Previous studies reported that the anatomical joint line should be located 10 mm proximal to the fibular head [15], suggesting that the prostheses were averagely located at an optimal position in our series. Elevation of the joint line should be limited to 5 mm to avoid patella baja and its associated adverse effects, such as anterior knee pain, ROM restriction, patellofemoral dysfunction, and mid-flexion instability [23, 24]. Our study showed that the postoperative joint line was reconstructed on average 11 mm above the fibular head using average thickness of 11 mm tibial inserts and 9 mm metal blocks with stem extension. Significant improvements were observed postoperatively in maximum extension angle from −10 ° to −1 °, ROM from 101 ° to 115 °, and KSS (knee score/function score) from 35/18 to 90/63. However, a case of knee instability (4 %) required exchange of the tibial polyethylene insert in our series. Rodriguez-Merchan previously reported that 10 to 22 % of TKA revisions were aimed at correcting instability [25]. Therefore, careful attention to soft tissue balance during TKA and late-onset instability at follow-up examination must be paid in RA patients with advanced forms of knee joint destruction. Cases of posterior subluxation in TKA-treated RA knees with flexion contracture were reported in the literature [12], but no knees showed dislocation in our series.

Radiolucent lines were detected beneath the metal blocks in 62 % (*n* = 16) of the knees treated with metal block augmentation for peripheral deficits. These radiolucent lines were not progressive and no loosening of the metal blocks was noted during the follow-up period. Fehring et al. reported that wedge-shaped augmentation was more favorable than block-shaped augmentation for bone stock preservation, although blocks were more advantageous for load bearing because they allowed for a planar bone-implant interface [26]. Tsukada et al. recently reported that radiolucent lines beneath the metal block-shaped augmentation were observed in 30 %, but no subsidence or loosening of the tibial tray was observed at 4 years after TKA [27]. Longer length of stem extension (100-mm vs. 40-mm or 60-mm) and follow-up period (6 years vs. 4 years) could affect higher proportion of patients with radiolucent lines in our study (62 %). And furthermore, cementation technique omitting vacuum mixing and pulsed lavage also could explain the larger number of radiolucencies [28, 29], although previous studies have not provide information regarding cementation technique (e.g. tourniquet use, type and viscosity of cement, vacuum mixing, pulsed lavage, hand packing, etc.) [14, 27, 30]. Pagnano et al. previously reported that radiolucent lines at the cement bone interface beneath the tibial wedge augmentation were present in 50 % [30]. Yamanaka et al. recently reported no failure of the metal wedge augmentation even for RA knees with large tibial condyle defects 6.3 years after primary TKA [14]. Therefore, another study with comparison in the risk of radiolucent line or loosening between block-and wedge-shaped augmentations are needed.

In our series, 100-mm stem extensions were used to relieve mechanical stress imposed on the bone-implant interface in patients treated with metal augmentation [31]. Tibial offsets were used at a rate of 40 % and were helpful in achieving better bone coverage. Sah et al. also reported that offset stems were used in 51 % of revision TKA [18]. Tang et al. demonstrated that the mean tibial shaft offsets were located anterolateral to the center of the tibial plateau [32]. Heterogeneous osteosclerotic changes were noted in 54 % of the tibial bones. In half of these knees, nonprogressive radiolucent lines were observed. Cortical proliferative changes were noted at a rate of 10 % around the distal end of the tibial stem extensions. These changes were not associated with pain or knee scores. Barrack et al. showed that 15 to 20 % of individuals with knees treated with stem extensions perceived pain around the distal ends [33]. However, Sah et al. reported that end-of-stem pain occurred rarely in hybrid cementing technique with a diaphyseal engaging press-fit stem [18]. Further follow-up is needed to obtain more detailed information.

A case of supracondylar femoral fragility fracture and a case of traumatic supracondylar femur fracture occurred in the early and late postoperative courses, respectively. The risk of periprosthetic fragility fractures was highlighted for TKA knees in patients with RA [34, 35]. The risk factors for fragility fractures include long duration of RA [6, 7, 34], insufficient disease control [34], increase in the intensity of activities of daily living from the preoperative to postoperative periods, old age [35], female sex [35], steroid use [35], and severe deformity [7]. Consequently, it is necessary to carefully assess bone quality and quantity in severely damaged knees before TKA, using computed tomography, dual-energy x-ray absorptiometry, and other diagnostic methods. As RA frequently reduces bone rigidity, tight control strategies for RA itself and osteoporosis are recommended to improve bone quality. The use of stem extensions is also an effective option in patients with bone atrophy compromising implant fixation.

Our study has some drawbacks. First, we were certainly limited by the small number of patients with the high rates of knees lost to follow-up (31 %) and intermediate follow-up period (6 years on average). Although this cohort is similar to the previous studies, larger numbers of patients with longer term of follow-up are required. However, compared to the literatures including various types of prostheses [14, 27], examination of currently used one particular prosthetic design could be a strength of our study. Second, a control group was not included. Therefore, further investigations with comparisons between RA and OA patients are needed. Third, components were fixed with cement to the cut surface without vacuum mixing or pulsed lavage in this case series. Especially pulsed lavage could improve interface strength significantly while reducing radiolucent lines at the cement-bone interface [28, 29]. We currently use pulsed lavage on a routine base in TKA. Finally, a cohort of patients included some cases with TKA in the contralateral knee. However, Ranstam and Robertsson have shown that survival rates are not or very little affected by bilateral cases [36]. Thus, we believe that the inclusion of these cases does not fundamentally alter conclusions of this study.

## Conclusions

Metal block augmentation and stem extension are reliable and durable primary TKA tools for severely damaged RA knees characterized by large bone deficits. This study showed that these procedures effectively restored knee function with proper joint line and provide satisfactory clinical results for a mean postoperative period of 6 years. Larger numbers of patients with further follow-up are required to determine the long-term performances of TKA using metal block augmentation and stem extension.

## Abbreviations

TKA: Total knee arthroplasty
RA: Rheumatoid arthritis
OA: Osteoarthritis
ROM: Range of motion
FTA: Femorotibial angle
KSS: Knee Society Score
AORI: Anderson Orthopaedic Research Institute
CI: Confidence intervals
